# Efficacy of spironolactone as adjunctive therapy to sodium valproate in bipolar‐I disorder: A double‐blind, randomized, placebo‐controlled clinical trial

**DOI:** 10.1002/brb3.3313

**Published:** 2023-11-06

**Authors:** Atefeh Zandifar, Rahim Badrfam, Fatemeh Gholamian, Arman Shafiee

**Affiliations:** ^1^ Social Determinants of Health Research Center Alborz University of Medical Sciences Karaj Iran; ^2^ Department of Psychiatry, Imam Hossein Hospital, School of Medicine Alborz University of Medical Sciences Karaj Iran; ^3^ School of Medicine Alborz University of Medical Sciences Karaj Iran; ^4^ Clinical Research Development Unit Alborz University of Medical Sciences Karaj Iran; ^5^ Student Research Committee, School of Medicine Alborz University of Medical Sciences Karaj Iran

**Keywords:** bipolar disorder, cognition, hypothalamus–pituitary–adrenal axis, spironolactone

## Abstract

**Introduction:**

Treatment of mood and cognitive symptoms of patients with bipolar disorder is associated with many complications and is generally not associated with therapeutic satisfaction. In this clinical trial, we evaluated the effectiveness of spironolactone in controlling mood and cognitive symptoms, sleep quality, appetite, and body mass index in patients with bipolar disorder in manic episodes.

**Methods:**

Sixty inpatients with bipolar disorder in manic episodes were treated with spironolactone/placebo in an 8‐week randomized, double‐blind, placebo‐controlled clinical trial. They were evaluated using the Young Mania Rating Scale (YMRS), mini‐mental state examination (MMSE), Pittsburgh sleep quality index, Simplified Nutritional Appetite Questionnaire, and body mass index in weeks 1, 4, and 8.

**Results:**

For cognitive impairment (MMSE), there were significant interaction effects of group and time at week 8 (B = −1.60, SE = 0.69, *t* = −2.33, *p* = .021) such that individuals in the spironolactone group experienced more improvement in their cognitive performance. For manic symptoms (YMRS), there were no significant interaction effects of group and time at week 8 (B = −2.53, SE = 1.46, *t* = −1.73, *p* = .085).

**Conclusions:**

Considering the promising findings in this clinical trial, further study of spironolactone as adjunctive therapy in bipolar disorder in manic episodes with larger sample sizes, multicenter settings, and longer follow‐ups are recommended.

## 1 INTRODUCTION

Bipolar disorder is a persistent chronic and severe disorder characterized by changes in mood and activity (Steardo Jr et al., 2019; Toyoshima et al., 2019). It has a prevalence of 1%–2% of the world's population (Canzian et al., 2022). This disorder is significantly associated with a decrease in psychosocial functioning and an approximate reduction of 10 to 20 potential years of life (McIntyre et al., 2020). Bipolar disorder type 1 is characterized by distinct manic episodes (Barbuti et al., 2023).

Despite the widespread use of mood stabilizers and atypical antipsychotics in combination with cognitive behavioral therapy and psychosocial support to treat acute episodes and prevent the recurrence of symptoms in bipolar disorder, the overall effectiveness of these treatment methods is not satisfactory, especially in advanced cases of the disease (Singhal & Baune, 2021). Based on this, despite the availability of pharmacological and non‐pharmacological treatments for patients with bipolar disorder, a large number of these patients are symptomatic in different stages of the disease. Based on this, it seems that paying attention to the underlying neurobiology of this disorder is important for identifying new therapeutic targets (Madireddy & Madireddy, 2022; Scaini et al., 2020). Genome‐wide aspects and epigenetic evidence emphasize attention to more objective components such as inflammation, oxidative stress, hypothalamus–pituitary–adrenal (HPA) axis changes, circadian rhythm problems, and nutritional issues as worthy components of therapeutic interventions in this field (Wartchow et al., 2023).

The association of cognitive deficits with bipolar disorder and their persistence beyond the improvement of acute episodes and independent of adequate treatment of the underlying mood disorder have been the focus of scientists in this field in the last two decades (Lima et al., 2019). Cognitive impairment with an intensity corresponding to the clinical status of mood episodes occurs in most people with bipolar disorders in manic and depressive episodes and continues during the euthymic periods of these patients (Reininghaus et al., 2020; Vanderplow et al., 2021). According to our knowledge, so far, no effective treatment, especially in the psychopharmacological field, has been reported that shows a robust and enduring effect on the cognitive impairment of bipolar disorder.

On the other hand, according to some studies, there is a relationship between sleep problems and cognitive impairment among patients with bipolar disorder (Laskemoen et al., 2020). Therefore, we proposed such a hypothesis that the use of medication with possible positive effects on the sleep quality of patients with bipolar disorder may be accompanied by the improvement of cognitive status in these patients.

Also, patients with bipolar disorder experience a higher prevalence of obesity and metabolic syndrome (Bora et al., 2019). In a comparative study between patients with unipolar depression and bipolar disorder, under conditions of controlled and identical age and gender distribution, patients with bipolar disorder had higher body mass index (BMI) compared to patients with unipolar depression (Bai et al., 2020).

Mineralocorticoid receptor (MR) belongs to a class of steroid hormone receptors that regulate the transcription of genes related to sodium reabsorption and potassium excretion and, in turn, are regulated by the renin–angiotensin–aldosterone system (RAAS) (Kanki & Young, 2021). Spironolactone, which is a MR antagonist ( Chen et al., 2020), is approved for the management of heart failure, hypertension, and edema, and due to its role as an antagonist of androgen receptors (Gabbard et al., 2020) and its aldosterone receptor antagonist properties (Aguilar Medina et al., 2022) is also used off‐label to manage conditions such as acne, hidradenitis, androgenetic alopecia, and hirsutism; and there is evidence in favor of its effectiveness in prostate cancer (Bommareddy et al., 2022). Its anti‐androgenic properties may also play a role in improving unwanted and disturbing psychological symptoms in women with excessive androgen production (Searle et al., 2020). The active metabolite of spironolactone, called canrenone, crosses the blood–brain barrier fairly well (Luft, 2016).

In a case series to investigate the effect of spironolactone on residual symptoms of bipolar disorder, four cases of euthymic bipolar disorder were evaluated with spironolactone as adjuvant treatment. Clinical response to residual symptoms and improvement of stress response were seen in all four patients. Such an effect was attributed to the antagonistic effects of mineralocorticoid receptors by spironolactone (Juruena et al., 2009). Also, in an old case series, among six patients with bipolar disorder under maintenance treatment with lithium who requested to stop treatment due to side effects of lithium, the use of spironolactone for 12 months was associated with the maintenance of conditions among five of them. The same effectiveness of spironolactone and lithium in suppressing aldosterone hormone fluctuations and their effect on biogenic amines, creating electrolyte stability, and controlling membrane fluctuations was mentioned as an effective factor in maintaining the mood stability of these patients (Hendler, 1978). In an animal study, inhibition of components of the RAAS was associated with the reversal of behavioral and/or cognitive symptoms in animal models with mood disorders such as bipolar disorder (Mohite et al., 2020).

Therefore, in addition to being related to neuro‐immunological processes and regulation of fluid and electrolyte balance, RAAS may be related to psychiatric conditions including mood disorders. The role of mineralocorticoid receptors in circadian rhythm regulation has also been considered in recent studies (Kanki & Young, 2021). Also, based on some evidence related to animal studies, this electrolyte regulation may be associated with benefits in terms of body mass status (Pfalzgraff & Skov, 2022).

Based on this, the use of medication with the simultaneous effect on the mood and cognitive status of patients with bipolar disorder, which also can influence the quality of sleep and control the metabolic and nutritional status of these patients, may be associated with clinical benefits in them. According to our knowledge, the effectiveness of mineralocorticoid receptor antagonists as an adjunctive treatment on the mood and cognitive status of patients with bipolar disorder has not been evaluated in any clinical trial.

Our hypothesis in this study is the effectiveness of spironolactone as an MR antagonist with anti‐inflammatory and HPA axis regulatory properties on the mood and cognitive symptoms of patients with bipolar disorder in manic episodes. Also, considering the regulatory feature of this medicinal treatment on electrolyte balance and circadian rhythm, we investigated its effect on the body mass index and sleep quality of these patients.

## 2 MATERIALS AND METHODS

This study, as a randomized, double‐blind, parallel‐group clinical trial, evaluated the effect of adjunctive treatment with spironolactone compared to placebo combined with one of the main types of the treatment of bipolar disorder type 1 in manic episodes (sodium valproate) for 8 weeks. It was conducted between May 2021 and November 2021.

### 2.1 Participant

The statistical population included patients referred to the outpatient clinic of Imam Ali Hospital, Alborz, Iran, who needed hospitalization in the psychiatric ward due to the severity of their symptoms. They were then admitted to the psychiatry department of Imam Hossein Hospital, Alborz, Iran. After conducting an independent structured interview based on the criteria of the Diagnostic and Statistical Manual of Mental Disorders—Fifth Edition (DSM‐5) by two experienced psychiatrists who were faculty members of the Psychiatry Department of Alborz University of Medical Sciences, in case of the final diagnosis of bipolar disorder type 1 in the manic episode and providing written consent by the patient's guardian, they were included in the study.

The study inclusion criteria included patients diagnosed with bipolar disorder type 1 in a mania episode, who were between the ages of 18 and 50 and had a minimum score of 20 based on the Young Mania Rating Scale (YMRS). It was also necessary to provide a written consent form from the patients and/or their guardians to enter the study. Exclusion criteria included comorbidity of obsessive‐compulsive disorder, anxiety disorders, any substance use during the 3 months before the recent episode (except for nicotine and caffeine use in the absence of any dependence), non‐continuity of participation in the study, accompanying neurological or cardiovascular disorders at the same time, accompanying any metabolic and hormonal disorder in need of medication, active kidney and liver disease, presence of any chronic disease, history of malignancy, immune deficiency, major surgery, any chronic medical disorder requiring long‐term medication, any fluid–electrolyte disorder, hyperkalemia, acidosis, pregnancy or breastfeeding, history of allergy to spironolactone and other drugs in the same category, and simultaneous use of steroids, lithium, and any drug that increases serum potassium levels (including angiotensin‐converting enzyme inhibitors, angiotensin receptor antagonists, digoxin, cholestyramine, NSAIDS, eplerenone, heparin, skeletal muscle relaxants such as cyclobenzaprine, diuretics such as amiloride, norepinephrine, potassium supplements, and other related drugs).

### 2.2 Measures

#### 2.2.1 Young mania rating scale

The YMRS is one of the most reliable and widely used tools for measuring the severity of manic episodes, which consists of 11 items. Evaluations are made through the patients' statements regarding their clinical condition during the last 48 h before the interview and also through the observations made during the interview by the clinician. The reliability, validity, and sensitivity of this questionnaire have been well evaluated (Young et al., 1978). Four items (irritability, speech, thought content, and disruptive/aggressive behavior) are graded based on a scale of 0 to 8, and the rest of the items (elevated mood, increased motor activity–energy, sexual interest, sleep, language–thought disorder, appearance, insight) are graded based on a scale of 0 to 4 (Lukasiewicz et al., 2013).

In evaluating the psychometric properties of this questionnaire in Iran, in a study in both clinical and non‐clinical populations, Cronbach's alpha coefficient was reported as .72, and its cut‐off point, sensitivity, and specificity were reported as 12.5, 0.93, and 0.96, respectively, which indicates its acceptability for use in Iranian population (Mohammadi et al., 2018).

#### 2.2.2 Mini‐mental state examination

The Mini‐Mental State Examination (MMSE) is used as a tool for brief screening and determining the severity of cognitive changes over time (Tombaugh & McIntyre, 1992). This tool evaluates cognitive performance in the fields of attention and orientation, memory, registration, recall, calculation, language, and the ability to draw a complex polygon by registering a maximum of 30 points (Arevalo‐Rodriguez et al., 2015).

A study in Iran evaluated the reliability and validity of the MMSE and described it as satisfactory (Cronbach's alpha coefficient equal to 0.78). This study reported the sensitivity and specificity of the above tool at the cut‐off point of 21, 90%, and 84%, respectively, in the population under study (Foroughan et al., 2008).

#### 2.2.3 Pittsburgh sleep quality index

The Pittsburgh Sleep Quality Index (PSQI) is widely used in different populations as one of the best tools to measure sleep quality during the month before evaluation. This self‐assessment questionnaire has 7 subscales (subjective sleep quality, sleep latency, sleep duration, habitual sleep efficiency, sleep disturbances, use of sleeping medication, and daytime dysfunction) and 19 individual items, where each item has a 4‐point Likert scale from 0 to 3 (Buysse et al., 1989; Zhang et al., 2020).

In a study in Iran among psychiatric patients along with the control group to evaluate the reliability and validity of the Persian version of this questionnaire, Cronbach's alpha coefficient for all subjects, the patient group, and the control group were reported as 0.77, 0.52, and 0.78, respectively. Also, the sensitivity and specificity of the questionnaire at cut‐off point 5 were 94% and 72%, respectively, which is acceptable for use in the Iranian population (Farrahi Moghaddam et al., 2012).

#### 2.2.4 Simplified nutritional appetite questionnaire

Simplified Nutritional Appetite Questionnaire (SNAQ) as a simple tool to assess appetite and predict weight loss, has a variable total score between 4 and 20, and the cutoff of ≤14 is considered a predictor of malnutrition and involuntary weight loss (Lau et al., 2020; Wilson et al., 2005).

The internal consistency of the Persian version of this questionnaire was confirmed based on Cronbach's coefficient of .7. Also, based on Pearson's correlation, the test–retest reliability of the questionnaire with a 2‐week interval was reported as .85 (Mohammadi et al., 2019).

#### 2.2.5 Body mass index

Body mass index (BMI) is used as a number expressing the anthropometric characteristics of height/weight in adults and is obtained from the result of mass(kilogram)/height(meter)^2^ (Burton, 2007; Nuttall, 2015). Typically, this quantitative index is used to define a person as underweight, normal weight, overweight, or obese (respectively with the cut‐off point of under 18.5 kg/m^2^, greater than or equal to 18.5 to 24.9 kg/m^2^, greater than or equal to 25 to 29.9 kg/m^2^, and greater than or equal to 30 kg/m^2^) (Weir & Jan, 2019).

### 2.3 Procedures

#### 2.3.1 Outcome

The primary aim of this study was to investigate the effect of spironolactone as adjunctive therapy compared to placebo on reducing the severity of mania symptoms in patients with bipolar 1 disorder. Patients referred to the outpatient clinic of Imam Ali Hospital with the diagnosis of bipolar disorder type 1 in a mania episode were evaluated to enter the study. These patients were subjected to additional examinations in terms of clinical, psychiatric, and pharmacological conditions if they met the inclusion criteria and did not meet the exclusion criteria (along with obtaining informed consent from the patient/patient's guardian).

They were examined in terms of the severity of mania symptoms using YMRS, the severity of cognitive impairment using MMSE, and quality of sleep using PSQI at the beginning of the study and then in weeks 4 and 8. Also, the appetite status of the patients was evaluated using SNAQ, and the condition of body mass was examined using BMI at the beginning and end of the study. They were also regularly evaluated using the side‐effect checklist in terms of medication side effects related to the clinical trial.

#### 2.3.2 Intervention

After screening and final identification of patients participating in the study, they were randomly assigned to two groups receiving spironolactone or placebo along with receiving sodium valproate as the main mood stabilizer, based on the alternative block randomization method. In one group, the patients were prescribed spironolactone at a dose of 50 mg daily for 8 weeks, together with sodium valproate at a dose of 200 mg 3 times a day at an interval of 8 h, and in the other group, instead of spironolactone, a placebo of the same shape, size, and color was used once a day. None of the patients, psychiatrists, and pill providers knew about the content of the pills and were also unaware of the placement of the patients in each group. Before starting the study, all patients were subjected to initial clinical examinations, evaluation of vital signs, and basic electrolyte and metabolic tests. All these steps were carried out by and under the supervision of two experienced psychiatrists and faculty members of the Department of Psychiatry of Alborz University of Medical Sciences.

#### 2.3.3 Randomization, allocation concealment, and blinding

The participants in the study were randomized into two groups receiving adjunctive treatment of spironolactone or placebo using a computer random generator as an unpredictable random method. A person who was not a member of the research team put the pills, which were the same in shape, color, and size, into two groups in separate envelopes. This person did not know the nature of the pills. Using a table of random numbers, based on the number of participants in the study, numbers were typed on special cards with the same shape, and each card was placed in a medicine envelope. The medicine envelopes were prepared in a sealed form.

After the participants entered the study, each envelope was assigned to them based on the number written on it corresponding to the patient's number. During the implementation of the study until the time of discharge, the pills were given to the patient one daily in the morning by the special nurse who was unaware of the nature of them. After discharge, the rest of the pills were given to the patient's guardian to be given to the patient daily. The patient's guardian was also unaware of the type of drugs (spironolactone/placebo). Psychiatrists and patients also did not know about the nature of the pills. Based on this design, during the 8‐week implementation of the study, people who were not members of the research team randomly provided the pills to the patients.

#### 2.3.4 Ethical considerations

The authors declare that all the steps of conducting this study were by the ethical standards of the relevant national and institutional committees on human experimentation and the 1975 Declaration of Helsinki and its revisions in 2008.

The research ethics committee of Alborz University of Medical Sciences approved all procedures involving human subjects/patients in this study with the number of the code of ethics IR.ABZUMS.REC.1399.319 and the registration code number IRCT20190316043072N2 are related to the registration of the study protocol of Iranian clinical trials. The registration date of this study is April 29, 2021.

### 2.4 Data analysis

#### 2.4.1 Power analysis

Considering beta 20% and alpha 5%, the required sample size for this clinical trial was considered to be 50 patients, and assuming 10% attrition during the study and to increase the power of the study, 60 patients were estimated as the required sample size.

#### 2.4.2 Statistical analysis

The description of quantitative and qualitative variables was based on the mean (standard deviation) and number (percentage), respectively. IBM SPSS Statistics 25 was used for conducting these analyses, and R statistical software with packages lme4 and lmerTest was used to estimate multilevel linear mixed effect models to determine if the treatment group predicted changes in outcome measures (Bates et al., 2014; Kuznetsova et al., 2017). A restricted maximum likelihood estimate was utilized for missing data, and Feingold's approach was used to quantify effect sizes (Feingold, 2009, 2015).

## 3 RESULT

**FIGURE 1:**
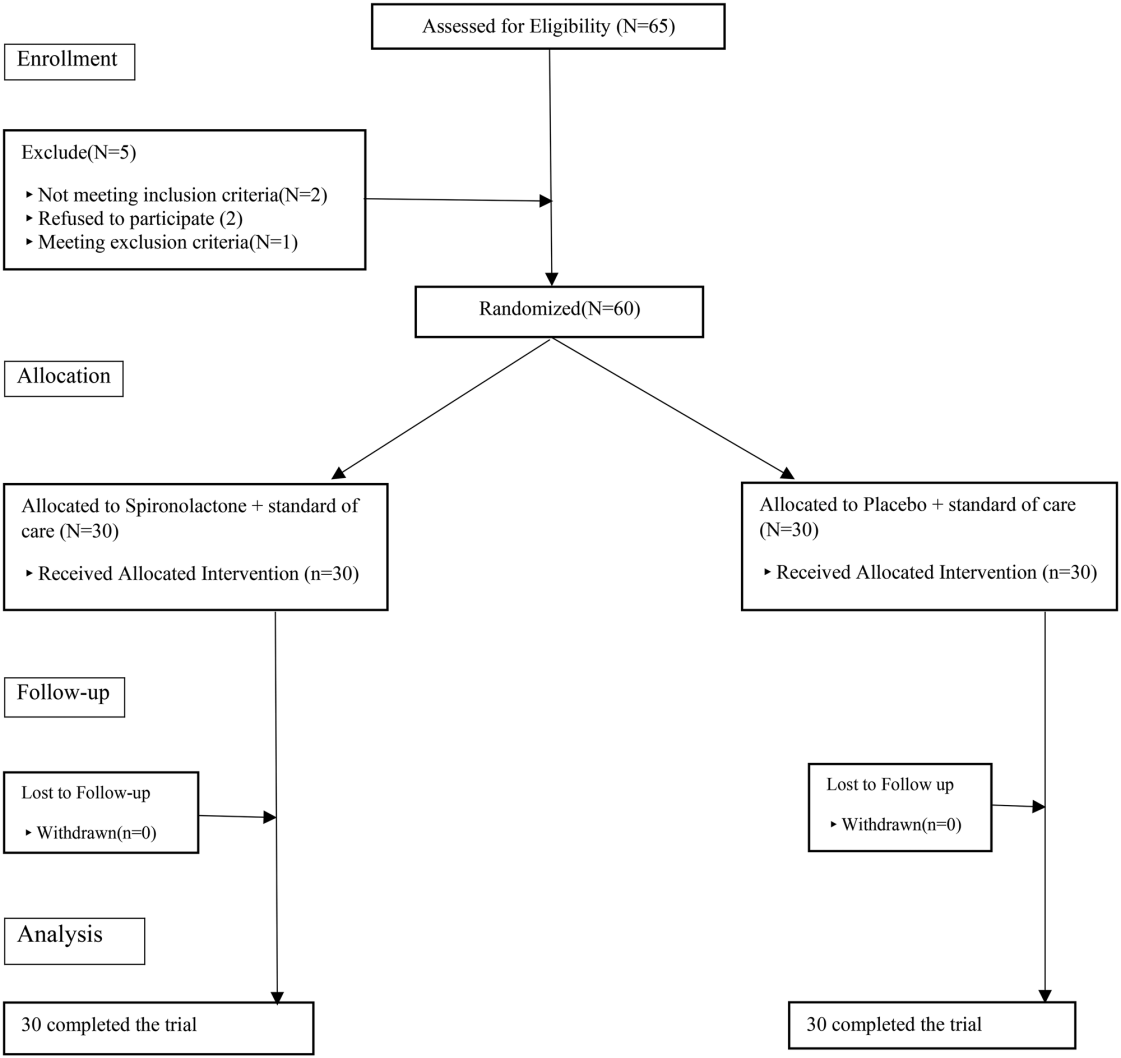
Flow diagram of the clinical trial of the efficacy of spironolactone as an adjunctive therapy on mania symptoms, cognitive impairments, sleep quality, and appetite of patients with bipolar disorder type 1.

The present study was conducted on 60 patients with bipolar disorder in the manic episode (Figure 1). After entering the study, the patients were divided into two groups of 30 people, treated with spironolactone and placebo (plus standard of care). The average age of the study participants in the group receiving spironolactone was 34.27 (± 12.19) years and in the group receiving placebo was 31.33 (± 10.62) years. Thirty‐nine patients (65%) of all participants in the study were women and 49 (81.7%) of them had a history of psychiatric hospitalization. Other demographic information of the patients is presented in Table 1.

**TABLE 1:** Background and demographic characteristics of patients.

		Number (%)/Average (standard deviation)			Chi^2^/*t*‐test	*p* Value
		Group A *N* (%)	Group B *N* (%)	Total		
Gender	Female	19 (63.3)	20 (66.6)	39 (65)	0.073	0.787
	Male	11 (36.6)	10 (33.3)	21 (35)		
Marital status	Single	11 (36.7)	16 (53.3)	27 (45)	1.926	0.382
	Married	15 (50)	10 (33.3)	25 (41.7)		
	Divorced	4 (13.3)	4 (13.3)	8 (13.3)		
Education	Primary school	1 (3.3)	1 (3.3)	2 (3.3)	2.887	0.577
	Middle school	3 (10)	3 (10)	6 (10)		
	High school	22 (73.3)	24 (80)	46 (76.7)		
	Bachelor's degree	4(13.3)	1 (3.3)	5 (8.3)		
	Doctorate	0 (0)	1 (3.3)	1 (1.7)		
History of psychiatric admission	Have	23 (76.7)	26 (86.7)	49 (81.7)	1.002	0.317
	Don't have	7 (23.3)	4 (13.3)	11 (18.3)		
Age (years)		34.27 (±12.19)	31.33 (±10.62)	32.80 (11.43)	0.993	0.325

Abbreviations: Group A, spironolactone users; Group B, placebo users.

For cognitive impairment (MMSE), the scores at the beginning of the study in the group receiving spironolactone and placebo were 21.6 (20.8–22.3) and 20.8 (20.1–21.6), respectively, which was a difference of 0.8. At the end of the study in the eighth week, these scores were 26.8 (26.1–27.6) and 24.5 (23.8–25.2), respectively, which was a 2/3 difference, and this difference was statistically significant in such a way that there were significant interaction effects of group and time at week 8 (B = −1.60, SE = 0.69, *t* = −2.33, *p* = .021) such that individuals in the spironolactone group experienced more improvement in their cognitive performance. This was while in the fourth week of the trial, despite the greater improvement in the cognitive status among patients receiving spironolactone compared to the group receiving placebo (average score 23.5 vs. 21.4), no statistically significant difference was found between the two groups (Group x time 2: B = −1.33, SE = 0.69, *t* = −1.94, *p* = .054).

For manic symptoms (YMRS), there were no significant interaction effects of group and time at week 8 (B = −2.53, SE = 1.46, *t* = −1.73, *p* = .085). For sleep quality (PSQI), there were significant interaction effects of group and time at week 4 (B = 1.16, SE = 0.58, *t* = 1.99, *p* = .048) such that individuals in the spironolactone group experienced greater decreases in sleep problems but there were no significant interaction effects of group and time at week 8 (B = 1.13, SE = 0.58, *t* = 1.94, *p* = .054). Between the two groups, there were no significant differences in terms of appetite (SANQ) or BMI. Tables 2 and 3 include the full model results.

**TABLE 2:** Results from linear mixed effects models.

Model	Parameter	B	SE	*t*	*p*
Appetite					
(SNAQ)	Intercept	12.53	0.53	23.68	<.001
	Time 3	0.03	0.30	0.11	.912
	Group	0.03	0.75	0.04	.965
	Group x time 3	−0.26	0.42	−0.63	.532
Body mass					
(BMI)	Intercept	24.07	0.44	55.86	<.001
	Time 3	−0.10	0.20	−0.49	.623
	Group	0.06	0.62	0.10	.915
	Group x time 3	0.20	0.29	0.70	.488
Mental status					
(MMSE)	Intercept	21.56	0.36	58.90	<.001
	Time 2	1.90	0.49	3.91	<.001*
	Time 3	5.26	0.49	10.84	<.001*
	Group	−0.73	0.52	−1.42	.158
	Group x time 2	−1.33	0.69	−1.94	.054
	Group x time 3	−1.60	0.69	−2.33	.021*
Mania					
(YMRS)	Intercept	46.53	0.90	51.70	<.001
	Time 2	−21.57	1.03	−20.89	<.001*
	Time 3	−29.07	1.03	−28.77	<.001*
	Group	4.00	1.27	3.14	<.001*
	Group x time 2	−3.66	1.46	−2.51	.013*
	Group x time 3	−2.53	1.46	−1.73	.085
Sleep difficulties					
(PSQI)	Intercept	17.70	0.40	44.18	<.001
	Time 2	−6.10	0.41	−14.77	<.001*
	Time 3	−9.06	0.41	−23.24	<.001*
	Group	−0.06	0.56	−0.12	.906
	Group x time 2	1.16	0.58	1.99	.048*
	Group x time 3	1.13	0.58	1.94	.054

*Note*: The intercept represents Week 0. Time 2 denotes the change from Week 0 to Week 4 across groups. Time 3 denotes changes from Week 0 to Week 8 across groups. Group denotes differences between the two treatment groups across time. Group x time 2 denotes the effect of the group on change from Week 0 to Week 4. Group x time 3 denotes the effect of the group on change from Week 0 to Week 8. Groups are coded 0= placebo, 1 = spironolactone.

Abbreviations: BMI, body mass index; MMSE, mini‐mental state examination; PSQI, Pittsburgh sleep quality index; SNAQ, Simplified Nutritional Appetite Questionnaire; YMRS, Young Mania Rating Scale.

**TABLE 3:** Marginal means from linear mixed effects models (95% CI).

Variable	Timepoint	Spironolactone (*n* = 30)	Placebo (*n* = 30)
		M (95% CI)	M (95% CI)
Appetite (SNAQ)			
	Week 0	12.5 (11.5–13.6)	12.6 (11.5–13.6)
	Week 8	12.6 (11.5–13.6)	12.3 (11.3–13.4)
Body mass (BMI)			
	Week 0	24.7 (23.8–25.6)	24.8 (23.9–25.6)
	Week 8	24.6 (23.7–25.5)	24.9 (24.0–25.7)
Mental status (MMSE)			
	Week 0	21.6 (20.8–22.3)	20.8 (20.1–21.6)
	Week 4	23.5 (22.7–24.2)	21.4 (20.7–22.1)
	Week 8	26.8 (26.1–27.6)	24.5 (23.8–25.2)
Mania (YMRS)			
	Week 0	46.5 (44.8–48.3)	50.5 (48.8–52.3)
	Week 4	25.0 (23.2–26.7)	25.4 (23.5–27.1)
	Week 8	16.8 (15.1–18.6)	18.3 (16.5–20.1)
Sleep difficulties (PSQI)			
	Week 0	17.7 (16.9–18.5)	17.6(16.8–18.4)
	Week 4	11.6 (10.8–12.3)	12.7(11.9–13.4)
	Week 8	8.1(7.3–8.8)	9.1 (8.3–9.9)

Abbreviations: BMI, body mass index; MMSE, mini‐mental state examination; PSQI, Pittsburgh sleep quality index; SNAQ, Simplified Nutritional Appetite Questionnaire; YMRS, Young Mania Rating Scale.

**FIGURE 3:**
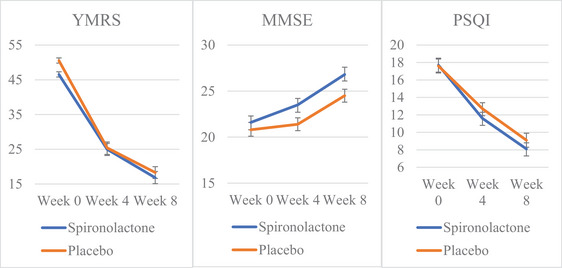
Graphical depictions of change over time in the spironolactone and placebo groups. YMRS, Young Mania Rating Scale; MMSE, mini‐mental state examination; PSQI, Pittsburgh sleep quality index.

The graphical representations of change over time in the spironolactone and placebo groups can be seen in Figures 2a–c and 3.

FIGURE 2(a) Changes in Young Mania Rating Scale (YMRS) scores based on the group: time. (b) Changes in mini‐mental state examination (MMSE) scores based on the group: time. (c) Changes in Pittsburgh sleep quality index (PSQI) scores based on the group: time.

**Figure:**
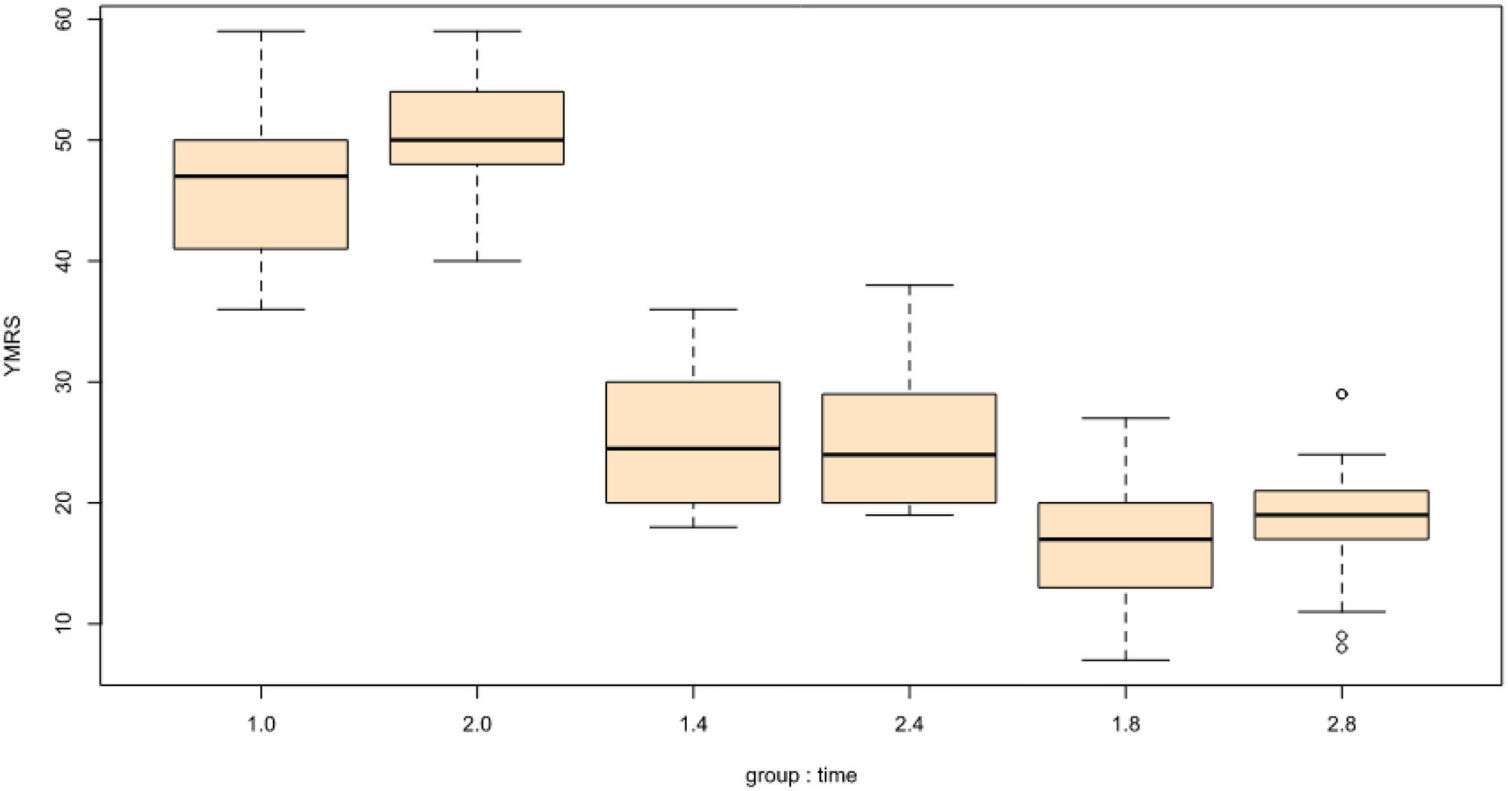

**Figure:**
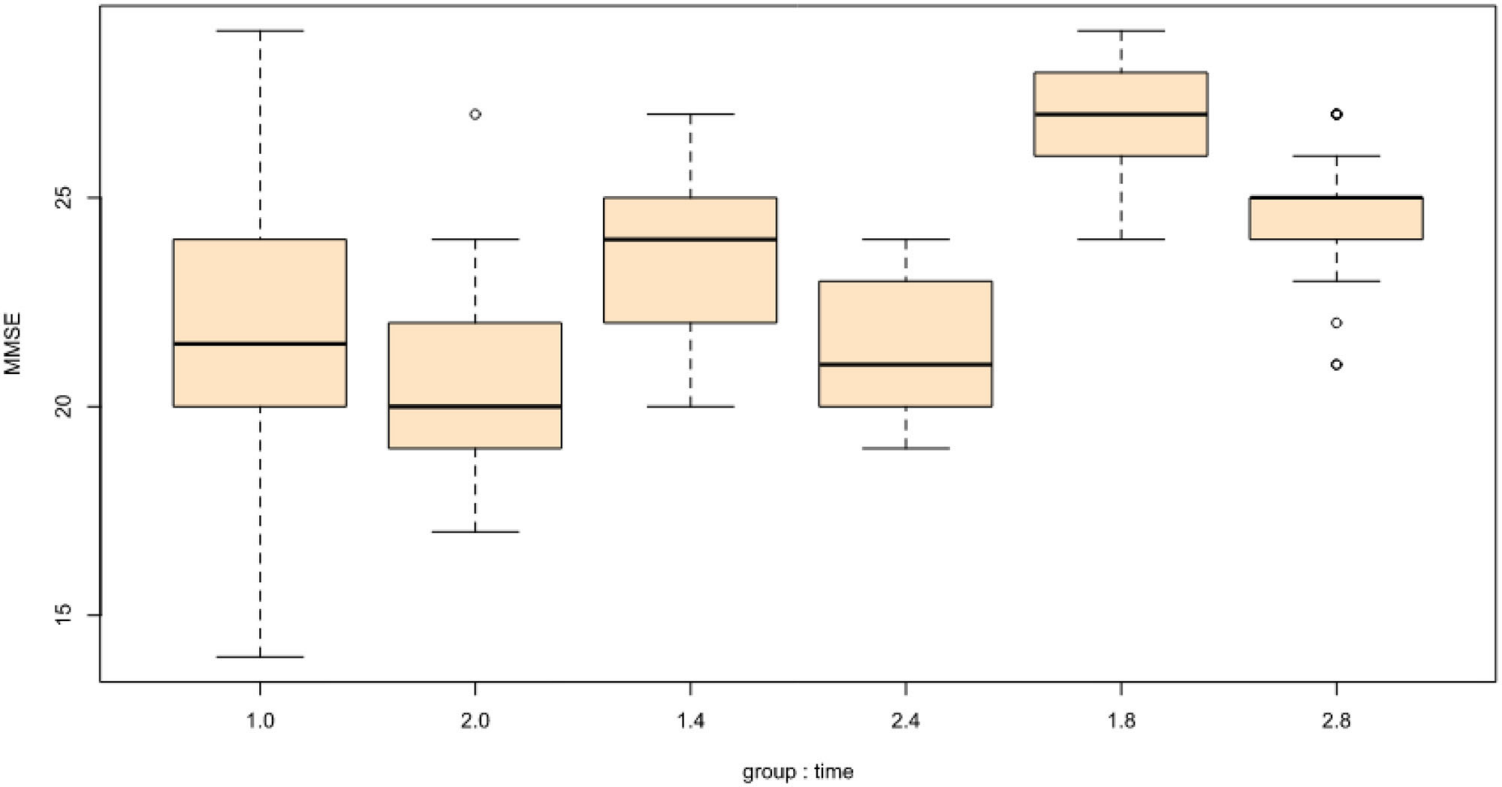

**Figure:**
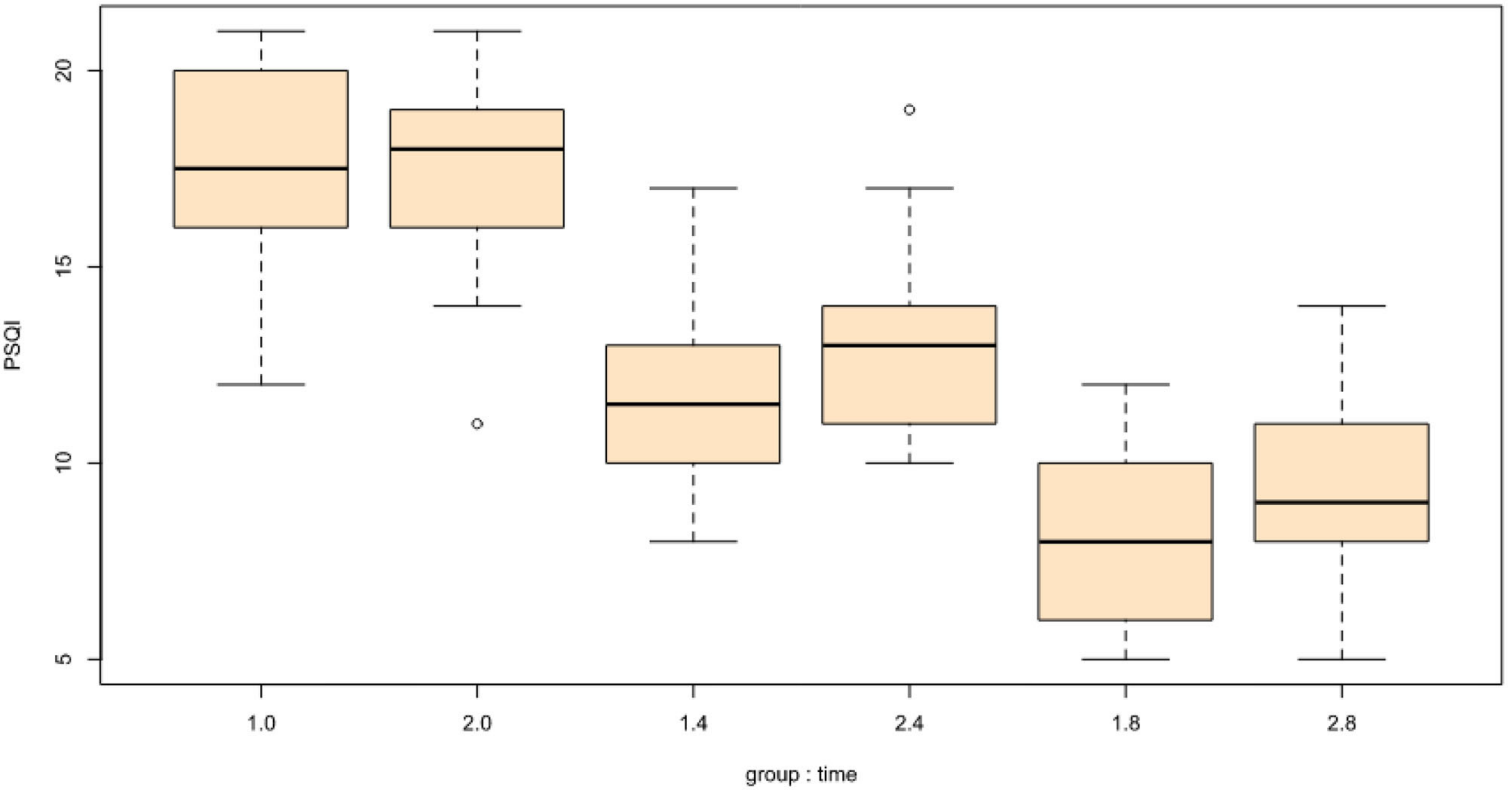

## 4 DISCUSSION

In this clinical trial, patients with bipolar disorder requiring hospitalization during a manic episode who received spironolactone showed a significant improvement in cognitive status compared to the placebo group at the end of 8 weeks of the study. This was while there was no statistically significant difference between the two groups receiving spironolactone and placebo in terms of other investigated variables such as mood state, sleep quality, appetite, and body mass index at the end of 8 weeks. No side effects were observed during the clinical trial. Based on our knowledge, this is the first clinical trial to investigate the usefulness of spironolactone as an MR antagonist, with all its systemic effects on fluid and electrolyte regulation, control of inflammatory status and oxidative stress, and regulation of the HPA axis and circadian rhythm on mood, cognition, sleep quality, and body mass index in patients with bipolar disorder in manic episodes.

Mood disorders are accompanied by chronic systemic inflammation and increased plasma levels of pro‐inflammatory cytokines such as tumor necrosis factor‐alpha (TNF‐α) and interleukin‐6 (IL‐6). These pro‐inflammatory factors regulate mood behavior and cognition by affecting the level of neurotransmitters and activating stress‐responsive endocrine axes (Bauer & Teixeira, 2021). The role of inflammation in the pathophysiology of bipolar disorder has been the focus and emphasis of experts due to the frequent evidence of altered levels of cytokines and acute phase proteins, pathological microglial activation, and changes in biogenic amine neurotransmitters whose expression is regulated by TNF‐α (Pereira et al., 2021). A significant increase in inflammatory markers such as C‐reactive protein (CRP) has been reported in first‐episode mania (Kapici et al., 2023), and some studies have emphasized the inflammatory pathophysiology of manic episodes. They have described a part of the effect of psychotropic medicines on the symptoms of mania due to the anti‐inflammatory effects of these treatments (Goyal et al., 2023).

The point emphasized in our study, as the first clinical trial that investigated the effect of spironolactone as an MR antagonist on the cognitive deficits of patients with bipolar disorder in manic episodes, is the effectiveness of this medication in improving the cognitive deficits of these patients after 8 weeks in comparison with the control group.

Extensive or selective persistent cognitive deficits in cognitive domains such as verbal memory, attention, executive function, and psychomotor speed are seen in a significant percentage of patients with bipolar disorders and are one of the important treatment goals in this group of patients to improve social and occupational performance, reduce social costs related to lost work productivity, and enhance the quality of life (Miskowiak et al., 2019; Tamura et al., 2022). Recently, cognitive status is considered to have an upward causal effect on functional outcomes in these patients (Ehrminger et al., 2021), and it is considered related to impaired psychosocial function and poor quality of life (Li et al., 2020). In this way, cognitive improvements have been accompanied by functional improvement in patients with bipolar disorder, and according to some evidence, these deficits will continue in the interval between episodes (Tsapekos et al., 2023). On the other hand, many of the main mood stabilizers used in the treatment of bipolar disorder, including lithium, sodium valproate, and antipsychotics can have negative cognitive consequences (Xu et al., 2020). Despite this, there is limited information about the causes of these defects, and emerging evidence indicates empirical links between cognitive defects and things such as increased inflammation and reduced neuroprotection (Van Rheenen et al., 2020). The nature of cognitive deficits in bipolar disorder is such that, based on some evidence, in addition to the association of these deficits with acute and chronic manifestations of bipolar disorder, there may be a connection between some of these deficits and early risk markers of bipolar disorder. This is an issue that emphasizes the need to pay more attention to the diagnosis and treatment of cognitive impairments in patients with bipolar disorder (Kjærstad et al., 2020).

A decrease in brain‐derived neurotrophic factor (BDNF) serum levels has been reported during the mania episode of patients with bipolar disorder, along with a decrease in executive function and verbal memory. Cognitive impairments can be associated with the aggravation of this decrease in blood level (Mora et al., 2019). Some studies have emphasized the role of mineralocorticoids on hippocampal neuronal survival and plasticity through MR receptors, and in a rat study, spironolactone was associated with upregulation of BDNF (Hameed, 2014). In a study on rats, the use of an MR inhibitor (spironolactone) was associated with the facilitation of BDNF‐tropomyosin receptor kinase B‐cAMP response element‐binding protein signaling pathway, which is considered part of the neuroprotective pathway (Balogh et al., 2020).

In a study with a mouse model of Alzheimer's cognitive impairment, the use of MR antagonists (spironolactone and eplerenone) was associated with improved cognitive status. The authors reported changes such as increased BDNF and decreased TNF‐α in the frontal cortex and hippocampus as the main factors involved in this process which may be associated with the activation of the Nrf2‐dependent antioxidant system and the reduction of neuroinflammation (Chen et al., 2020).

Also, there is a relationship between sleep abnormalities and cognition in patients with bipolar disorder (Burgess et al., 2022). According to a study, sleep abnormalities in patients with bipolar disorder may be the main driver of cognitive dysfunction in these patients, and in this way, in patients with more sleep problems, a greater severity of cognitive impairments is expected (Bradley et al., 2020). Also, the treatment of sleep disorders has been proposed to protect cognitive function in these patients (Laskemoen et al., 2020).

In our study, the use of spironolactone was associated with the benefit and improvement of sleep quality at the end of the fourth week of the mood episode compared to the placebo, although the durability of this effect was not seen in the eighth week of the study.

Sleep problems in patients with bipolar disorder have a high prevalence, are highly distressing and destructive (Morton & Murray, 2020), and are seen across all phases and all age groups (Pal et al., 2022). Disturbance in the initiation and continuity of sleep and the decrease in its quality are some of the characteristics of patients with bipolar disorder, which are persistent in many cases during the inter‐episodic period (Meyer et al., 2020). These problems are highly prevalent in all stages of bipolar disorder, and the evidence from the most consistent findings in sleep electroencephalography studies in these patients shows a reduction in their overall need for sleep, independent of the stage of the disease (Zangani et al., 2020). In many cases, sleep abnormalities are a good predictor of mood swings in these patients, and for this reason, establishing stable sleep–wake cycles as an important factor in maintaining stability in patients with bipolar disorder is of special concern (Steardo Jr et al., 2019). Some studies have stated improving sleep is a potential early prevention target in patients with bipolar disorder (Hensch et al., 2019). Also, sleep problems are considered an important therapeutic focus (Morton & Murray, 2020) and these disturbances are potential targets for adjunctive interventions in patients with bipolar disorders (Bisdounis et al., 2022).

While the glucocorticoid receptor (GR) and its ligand, cortisol, are considered circadian clock regulators, according to new studies, MR also can play a role in circadian clock gene expression and timing regulation (Kanki & Young, 2021). It seems that investigating the effect of spironolactone on the sleep quality of patients with bipolar disorder, considering the mechanism of effect as an MR antagonist and its usefulness in the early stages of treating patients with bipolar disorder in manic episodes in our study, requires further evaluation in future studies using larger sample sizes and a longer duration of the clinical trial.

According to some studies, inflammation may be associated with weaker cognitive outcomes in bipolar disorder, and high CRP has been associated with some low cognitive indices in this group of patients (Millett et al., 2021). These inflammatory processes can effectively form cognitive impairments by disrupting the neurobiological mechanisms regulating cognition such as neurogenesis and affecting the HPA axis (Fourrier et al., 2019). The increase of inflammatory and pro‐inflammatory cytokines (TNFα, IL‐6, and IL‐1) is also a common finding in patients with bipolar disorder type 1, which is associated with poorer cognitive function, and is mentioned as a therapeutic strategy to maintain cognitive status in these patients (Chakrabarty et al., 2019).

Based on the studies conducted in patients with hypertension and diabetes, treatment with spironolactone is accompanied by a decrease in the serum levels of many inflammatory markers such as CRP and TNF‐α (Lin et al., 2021). Antioxidant effects of spironolactone have been reported in the form of reducing inflammatory and pro‐inflammatory processes (with markers such as decreasing levels of TNF‐α and interleukin 1 beta [IL‐1β]) (Velioğlu öğünç, 2019).

In a study on mice, the use of spironolactone as an antagonist of NRG1‐ERBB4 signaling was associated with the improvement of working memory impairment by reversing the hyperphosphorylation of activated Erbb4 (NRG1 and its cognate receptor ERBB4 are considered as schizophrenia risk genes and altered NRG‐ERBB4 signaling is associated with positive, negative, and cognitive symptoms) (Wehr et al., 2017). In another study on mice, the use of spironolactone was associated with cognitive benefit in reducing schizophrenia‐related reversal learning (Stephan et al., 2022).

In this way, an important part of the effect of spironolactone on the cognitive function of patients with bipolar disorder in the episode of mania in our study may be caused by the different mechanisms mentioned above.

Many psychiatric disturbances including bipolar disorder are deeply affected by the activity of the HPA axis, and at the same time, they also have a reciprocal effect on this neuroendocrine system (Trinetti et al., 2021). Based on growing evidence, an important part of the underlying psychopathology of bipolar disorder may be seen in persistent malfunction in the expression and role of mineralocorticoid and glucocorticoid receptors in the hippocampus, which mediates the effects of glucocorticoids in the form of negative feedback binding and is in a clear connection with the impact of stressful life events on the HPA axis (Juruena et al., 2021). Chronic stress mediated by the HPA axis—the subsequent increase in ACTH serum levels—may be related to the pathophysiology of bipolar disorder and especially manic symptoms (direct relationship between ACTH levels and manic symptoms) (Zhang et al., 2022).

Some studies reported increased hair cortisol concentrations as a sign of increased HPA activity in manic episodes of bipolar disorder(van den Berg et al., 2020). Such a situation was also reported in the nocturnal salivary cortisol level, in which a disturbance in the usual reduction of nocturnal cortisol was seen among patients with symptomatic bipolar disorder(Mukherjee et al., 2022). Based on the available evidence, HPA axis disturbance as the primary stress hormone system is also associated with resistance to treatment, increased relapse rates, worse outcomes, and the development of cognitive defects in patients with bipolar disorder (Juruena et al., 2022). Also, the use of glucocorticoid medication with an indirect effect on the HPA axis is associated with the occurrence of psychiatric symptoms such as mania (Alheira & Brasil, 2005).

On the other hand, systemic inflammation with damage to the blood–brain barrier and increasing its permeability causes the access of inflammatory mediators to the brain and local activation of glial cells and neuroinflammation, which continues with a series of disturbances in the regulation of the HPA axis, serotonin and melatonin neuroreceptor system, and neurogenesis (Hennion et al., 2021).

Based on the available evidence, the RAAS may play a role in regulating the HPA axis (Kakehi et al., 2023). Angiotensin can activate the HPA axis to increase circulating glucocorticoids, and ACTH is also effective in aldosterone production (El Ghorayeb et al., 2016; Grippo & Johnson, 2009).

In another clinical trial in which our group investigated the effect of spironolactone on the negative symptoms of patients with schizophrenia, the regulation of HPA activity by spironolactone was proposed as the main reason for the clinical effects of this medication treatment (Zandifar et al., 2022). Also, studies support the role of MRs in regulating the HPA axis in basal conditions as well as in response to stress (Cornelisse et al., 2011).

In a study by Hochman et al., the relationship between relative fluid retention and manic episodes in bipolar disorder was reported. They found a significant decrease in serum fluid balance indices during manic episodes compared to depressive episodes in the form of lower concentrations of hematocrit, sodium, and serum albumin (Hochman et al., 2014). In some studies, high aldosterone plasma levels have been reported in manic episodes of bipolar disorder (Akesode et al., 1976), and according to some studies, an increase in aldosterone during the switch from depression to mania has been seen in short‐cycle bipolar patients along with sodium retention (LEVELL & HULLIN, 2012). Some clinical case reports have also reported an increase in body water and a progressive increase in volume dilution without a known external reason (medication side effects and other influencing factors) in the manic episode (Rittmannsberger & Malsiner‐Walli, 2013). In addition to angiotensin 2 (Ang II), aldosterone release is also stimulated by ACTH and the sympathetic nervous system (Vian et al., 2017).

In this way, the use of spironolactone may improve the psychiatric symptoms of patients with bipolar disorder in manic episodes by creating fluid and electrolyte balance and reducing edema along with correcting the established state and preventing the reactions mentioned above. Also, the expression and function of GR and MR in the hippocampus are strongly regulated by serotonin (5‐HT) in the middle raphe nucleus, and stress is one of the effective factors in this receptor change through such a mechanism. The balance between these receptors in this center plays an important role in controlling emotional states (Wu et al., 2013). Such a mechanism emphasizes the role of spironolactone as an antagonist of mineralocorticoid receptors in mood control.

As mentioned, this result was not obtained at the end of 8 weeks of our study, and no difference was found in terms of the intensity of psychiatric symptoms between the two groups receiving spironolactone and placebo. In this way, conducting similar studies with larger sample sizes and using different doses of spironolactone may be beneficial in completing the present study.

On the other hand, gut microbial dysbiosis is effective in disease progression and cognitive impairment in bipolar disorder (Dai et al., 2022). In recent years, specific evidence has been obtained regarding the role of gut microbiome through the mediation of inflammation, with the formation and progression of bipolar disorder. This evidence has led to the development of approaches to using dietary, pro‐ and pre‐biotic interventions, as adjuvant treatments for this disorder (Gondalia et al., 2019). According to some evidence, the change in the natural microbiome following the use of antibiotics has been associated with an increased risk of inducing (hypo)mania in related case samples (with a possible mechanism related to the reduction of bacterial products such as gamma‐amino‐butyric acid) (Borkent et al., 2022). Microbiota–gut–brain axis has strong effects on controlling the synthesis of neurotransmitters such as serotonin and mediating the activation of the HPA axis (Ortega et al., 2023). Disorders related to gut microbiota can be effective in the formation of some neuropsychiatric disorders such as bipolar disorder through the production of biological products that are mediated through this axis (Mitrea et al., 2022). According to various studies, there is a direct relationship between the gut microbiota and the HPA axis, along with a clear relationship with the immune system, the gut barrier and the blood–brain barrier, microbial metabolites, and gut hormones (Farzi et al., 2018).

In a study on rats in a genetic model of neurogenic hypertension, spironolactone was associated with the improvement of intestinal dysbiosis. In this study, spironolactone restored the ratio of Firmicutes/Bacteroidetes and the population of acetate‐producing bacteria to normal levels and reduced the percentage of intestinal aerobic bacteria (González‐Correa et al., 2023). In this regard, in a study on mice, MR deficiency was reported to have a protective effect on induced colitis. This result was accompanied by vast changes in gut microbiota, and its positive effects were considered related to the reduction of inflammatory cytokine production and the reduction of infiltration of monocytes, neutrophils, and interferon γ+ T cells in the lamina propria of the colon (Liu et al., 2022).

In examining another aspect of the problems associated with bipolar disorder, points such as obesity, increased appetite, and weight gain can be mentioned due to underlying multifactorial reasons such as medication side effects, disturbances in the neuro‐endocrinal system, and other influencing factors such as genetic variants which have a high prevalence in these patients (Platzer et al., 2020). These patients are at increased risk of developing metabolic syndrome compared to the general population (Misiak et al., 2022). This syndrome is associated with changes in serum levels of glucose, cholesterol, and triglycerides along with blood pressure and anthropometric dimensions of the body (Fusar‐Poli et al., 2020). Also, eating disorders are one of the known comorbidities of bipolar disorder (Hirte et al., 2022; McDonald et al., 2019).

Ghrelin, as an appetite‐stimulating peptide, is associated with an increase in appetitive feeding behaviors and the frequency of eating meals. Such an effect occurs when ghrelin passes through the blood–brain barrier and affects body weight regulation centers such as the hypothalamus and the mesolimbic reward system (Cummings, 2006). According to some studies, patients with bipolar disorder have higher levels of plasma ghrelin compared to the control group (Chen et al., 2021). Although such a finding has not been directly reported about plasma leptin (an adipokine secreted by white adipose tissue in response to insulin) levels, its disproportionate increase with increasing BMI in bipolar disorder (in favor of the potential inflammatory role of white adipose tissue in this disorder) has been seen (Fernandes et al., 2016).

In a study in rats, the use of spironolactone as a metabolic modulator was associated with protection against lipid dysfunction (Areloegbe et al., 2022) and in an experimental study, the use of spironolactone with mineralocorticoid antagonistic effects compared to the control group was associated with a significant reduction in body mass loss following starvation only in the first week of use, while this effect was not different from the control group in the following weeks (Pfalzgraff & Skov, 2022).

The possible lack of influence of spironolactone on body mass index and the absence of side effects of weight gain can be associated with benefits in patients with bipolar disorder due to the inappropriate metabolic status of these patients. In our study, there was no significant difference in terms of body mass index and appetite between the two groups receiving spironolactone and placebo.

## 5 LIMITATIONS

The existence of clinical heterogeneity of bipolar disorder has led to the formation of various studies in the genetic, epigenetic, molecular, and cellular fields related to its pathophysiology and the provision of various diagnostic, preventive, and therapeutic suggestions related to it, which have also brought variable results (Wartchow et al., 2023). This path may be the basis for considering different sub‐group divisions among patients with bipolar disorder with variable responses to new treatment suggestions. This condition, while it can be the basis for further research, may evaluate the approaches that face some difficulties. The clinical response to the effect of spironolactone on the cognitive impairment of patients with bipolar disorder in manic episodes in our study and the absence of an evident and statistically significant response to the effect of this medication on other aspects measured in this study can be accompanied by such an interpretation and can be considered as part of the limitations of our study.

On the other hand, the average age of the participants in both spironolactone and placebo groups was between 30 and 35 years old. However, the baseline scores of MMSE were 21.6 for the spironolactone group and 20.8 for the placebo group which was too low considering their age. This point may be considered partly related to the effect of distraction and flight of ideas on manic symptoms and maybe only a temporary low score. We observed that as YMRS scores improved during weeks 4 and 8, MMSE scores also improved. The YMRS scores of the spironolactone group were 16.8 and those of the placebo group were 18.3 in week 8, and the slightly higher scores of the placebo group might be related to the lower improvement in MMSE scores. In this way, despite the statistical significance of the results obtained in the improvement of cognitive symptoms in the eighth week of this trial, it is possible to achieve a more accurate evaluation of these results by continuing to compare the results in subsequent studies after further improvement in YMRS scores in the two groups.

In this study, we used the standard therapeutic dose of sodium valproate (200 mg, 3 times a day at an interval of 8 h), the therapeutic usefulness of which we have experienced in our clinical experiences in this center. The use of sodium valproate plasma levels to control the treatment status of patients could be associated with benefits that were not possible in this study due to financial limitations.

Due to the severity of the mood and cognitive symptoms of the patients at the beginning of the evaluation of the participants in the study, there were limitations in data collection, which were managed by establishing proper communication with the patients and the cooperation of their caregivers and psychiatric nurses.

## 6 CONCLUSIONS

The treatment complications of bipolar disorder in manic episodes and the lack of satisfactory responses to usual pharmacological and non‐pharmacological treatments to control the episode and prevent the recurrence of symptoms emphasize the need to pay attention to the use of additional treatments. The use of spironolactone add‐on treatment as a mineralocorticoid receptor antagonist to sodium valproate in the manic episode of bipolar disorder among a group of hospitalized patients in this clinical trial was associated with an improvement in the cognitive status of these patients compared to placebo.

The multiplicity of different possible mechanisms influencing the improvement of the cognitive status of these patients (systemic effects on fluid and electrolyte regulation, control of inflammatory status and oxidative stress, and regulation of the HPA axis and circadian rhythm) emphasizes the necessity of designing related studies focusing on each of the possible influencing paths to achieve new methods of treating this disorder.

In this study, the usefulness of spironolactone‐added treatment to control mood symptoms, improve sleep quality, control appetite, and reduce mass index was not obtained. The need to pay attention to conducting similar studies using spironolactone as adjunctive therapy in the treatment of manic episodes of bipolar disorder using a larger sample size, multicenter setting, longer follow‐ups, and other possible doses of spironolactone is suggested.

## AUTHOR CONTRIBUTIONS

Conceptualization: Atefeh Zandifar, Rahim Badrfam, and Fatemeh Gholamian. Data curation: Atefeh Zandifar, Rahim Badrfam, Fatemeh Gholamian, and Arman Shafiee. Formal analysis: Atefeh Zandifar, Rahim Badrfam, and Arman Shafiee. Funding acquisition: Atefeh Zandifar. Investigation: Atefeh Zandifar, Rahim Badrfam, and Fatemeh Gholamian. Methodology: Atefeh Zandifar, Rahim Badrfam, and Fatemeh Gholamian. Project administration: Atefeh Zandifar, Rahim Badrfam, and Fatemeh Gholamian. Resources: Atefeh Zandifar, Rahim Badrfam, and Arman Shafiee. Software: Atefeh Zandifar, Rahim Badrfam, and Arman Shafiee. Supervision: Atefeh Zandifar and Rahim Badrfam. Validation: Atefeh Zandifar and Rahim Badrfam. Visualization: Atefeh Zandifar and Rahim Badrfam. Writing—original draft: Atefeh Zandifar and Rahim Badrfam. Writing—review and editing: Atefeh Zandifar and Rahim Badrfam. All authors reviewed and approved the final manuscript.

## CONFLICT OF INTEREST STATEMENT

All authors declare that they have no conflicts of interest.

### PEER REVIEW

The peer review history for this article is available at https://publons.com/publon/10.1002/brb3.3313

## Data Availability

The data that support the findings of this study are available from the corresponding author upon reasonable request.
